# The role of macrophage polarization in organ transplantation: research progress on impact on graft injury, repair, and fibrosis

**DOI:** 10.3389/fimmu.2026.1884206

**Published:** 2026-07-17

**Authors:** Liping Wang, Lijuan Wang, Xu Chen, Shaochen Yu

**Affiliations:** 1 Department of Infectious Diseases, Taizhou Central Hospital (Taizhou University Hospital), School of Medicine, Taizhou, Zhejiang, China; 2 Department of Respiratory and Critical Care Medicine, Taizhou Central Hospital (Taizhou University Hospital), School of Medicine, Taizhou, Zhejiang, China; 3 Department of Integrated Chinese and Western Medicine, Taizhou Central Hospital (Taizhou University Hospital), School of Medicine, Taizhou University, Taizhou, Zhejiang, China; 4 Department of Emergency and Critical Care Medicine, Chuzhou Integrated Traditional Chinese and Western Medicine Hospital, Chuzhou, Anhui, China

**Keywords:** fibrosis, graft injury, immunoregulation, macrophage polarization, organ transplantation, tissue repair

## Abstract

Organ transplantation is a life-saving therapy for patients with end-stage organ failure. However, post-transplant graft injury, immune rejection, and chronic fibrosis severely compromise the long-term survival of recipients and graft function. Macrophages, as core components of innate immunity, possess remarkable plasticity and can polarize into pro-inflammatory M1 or anti-inflammatory/reparative M2 phenotypes in response to microenvironmental signals, thereby deeply participating in the post-transplant immune response and tissue remodeling processes. This review systematically explores the regulatory network of macrophage polarization after transplantation and thoroughly analyzes the complex and often paradoxical roles of different polarization phenotypes in ischemia-reperfusion injury, acute and chronic rejection, graft repair, and progressive fibrosis. Based on current research progress, this article further envisions the therapeutic potential of precisely regulating macrophage polarization phenotypes to intervene in transplant-related pathological processes, aiming to provide innovative theoretical foundations and strategic directions for improving long-term graft survival and function.

## 1 Introduction

The success of organ transplantation depends not only on surgical techniques but also on a profound understanding of the complex post-transplant immune and repair processes. Macrophages, as tissue-resident or circulating monocyte-recruited crucial immune cells, serve as a bridge connecting innate and adaptive immunity. In the transplant setting, the ischemia-reperfusion injury (IRI) experienced by the graft, alloantigen recognition, and persistent immune attacks collectively constitute a dynamically changing microenvironment that profoundly influences the phenotype and function of infiltrating macrophages. Macrophage polarization—that is, their directed differentiation into different functional states such as classical activation (M1) or alternative activation (M2)—is a critical event determining transplant outcomes. M1 macrophages are typically induced by signals such as interferon-γ (IFN-γ) and lipopolysaccharide (LPS), secreting pro-inflammatory cytokines (e.g., TNF-α, IL-1β, IL-6), promoting Th1 immune responses, and exacerbating tissue injury and acute rejection (1). M2 macrophages, induced by IL−4, IL−13, IL−10, and other signals, can be further divided into subtypes: M2a (induced by IL−4/IL−13, involved in Th2 immunity and tissue repair), M2b (induced by immune complexes/TLR ligands, immunoregulatory), M2c (induced by IL−10/TGF−β/glucocorticoids, anti−inflammatory and pro−fibrotic), and M2d (induced by TLR/adenosine signals, angiogenic and tumor−promoting). These subtypes have overlapping yet distinct functions in transplantation (2). In recent years, novel technologies such as single-cell sequencing have revealed that macrophage heterogeneity in the transplant context far exceeds the traditional M1/M2 dichotomy, with the emergence of more functionally specialized subsets (2). However, the fate of the graft is ultimately determined by a more complex alloimmune response. Antigen-specific immune responses, triggered by HLA mismatches between donor and recipient, are central to acute rejection and chronic graft dysfunction. These responses not only drive macrophage polarization via T-cell-derived cytokines such as IFN-γ but also activate macrophages directly through antibody-mediated effector mechanisms. Therefore, a deep understanding of the crosstalk between macrophage polarization and antigen-specific alloimmunity is essential for developing effective therapeutic strategies. Therefore, a deep analysis of the precise regulatory network of macrophage polarization and its specific role in graft fate is crucial for the development of novel immune intervention strategies.

In the early post-transplant phase, IRI is an inevitable pathological process that activates the innate immune system by inducing cell death and releasing damage-associated molecular patterns (DAMPs). The liver, as the largest immune organ, has resident macrophages (Kupffer cells) that play a central role in IRI, while the infiltration of circulating monocytes and their differentiation into monocyte-derived macrophages further amplify the inflammatory response (3). It is important to distinguish between donor-derived tissue-resident macrophages (TRMs) and recipient-derived monocyte-derived macrophages (MDMs), as they differ fundamentally in origin, turnover, and functional responses. TRMs are established during embryogenesis and maintain themselves through self-renewal, typically exhibiting a quiescent, tissue-surveillance phenotype. In contrast, MDMs are recruited from the circulation in response to injury signals and exhibit high phenotypic plasticity, predominantly driving pro-inflammatory responses in the acute phase. This distinction has profound implications for understanding the relative contributions of these populations to graft injury, repair, and chronic fibrosis (4). Guided by microenvironmental signals, these infiltrating macrophages can polarize into pro-inflammatory M1 or anti-inflammatory/pro-repair M2 phenotypes. M1 macrophages exacerbate tissue injury by secreting factors such as TNF-α and IL-1β, whereas M2 macrophages participate in tissue repair and remodeling by secreting IL-10 and TGF-β (5). However, sustained activation of M2 macrophages can also lead to excessive repair, thereby promoting fibrosis. For instance, in systemic sclerosis (SSc)-associated skin fibrosis, IL-1β-activated microvascular endothelial cells can induce the differentiation of monocytes into DC-SIGN-positive alternatively activated macrophages, which produce high levels of CCL18 and promote fibroblast activation, closely correlating with the severity of skin fibrosis (6). Similarly, in renal fibrosis models, tissue transglutaminase (TG2) promotes the polarization of monocyte-derived macrophages towards M2 via the ALOX15 pathway, thereby exacerbating kidney fibrosis (7).

The regulatory network governing macrophage polarization is extremely complex, involving multiple signaling pathways and metabolic reprogramming. The JAK-STAT pathway is one of the core pathways regulating macrophage polarization. For example, IL-4/IL-13 drives M2 polarization by activating STAT6, while IFN-γ promotes M1 polarization via STAT1 (8). JAK inhibitors (Jakinibs) can simultaneously suppress the activation of both M1 and M2a macrophages, demonstrating dual anti-inflammatory and anti-fibrotic potential, providing new ideas for the treatment of diseases such as SSc-associated interstitial lung disease (9). Furthermore, alterations in metabolic pathways profoundly affect macrophage functional states. M1 macrophages rely on aerobic glycolysis, while M2 macrophages favor fatty acid oxidation and oxidative phosphorylation (10). Iron metabolism is another key regulatory node; chronic iron overload can induce monocyte-derived macrophages to polarize towards an M2-like phenotype (8), and ferroptosis is associated with M2 macrophage sensitivity, potentially participating in the formation of an immunosuppressive microenvironment (2, 11). These findings indicate that targeting specific signaling pathways or metabolic checkpoints could be an effective strategy for regulating macrophage polarization after transplantation.

**Figure 1:**
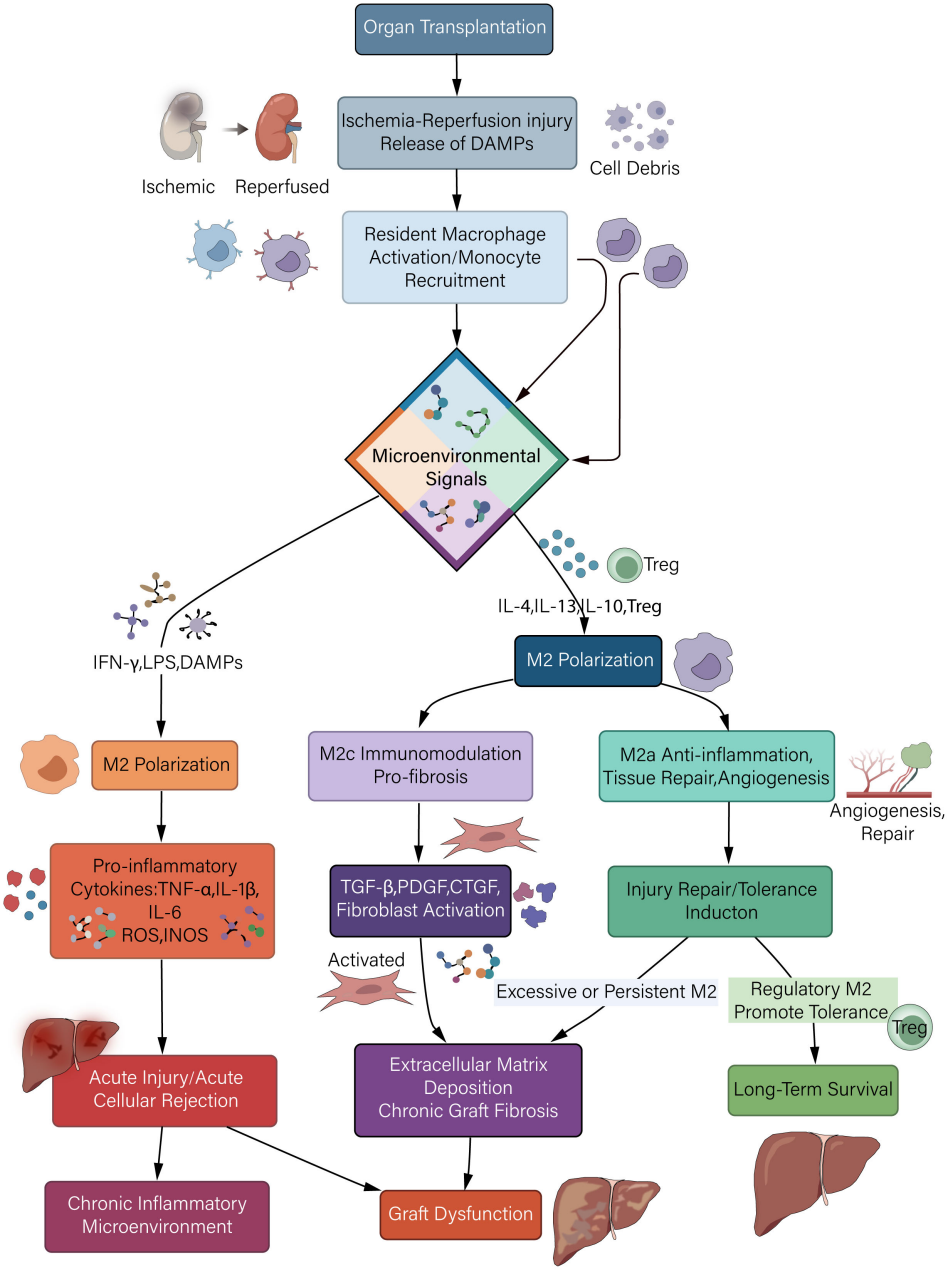
Overview of macrophage polarization dynamics and their impact on graft fate following organ transplantation. Following transplantation, ischemia-reperfusion injury (IRI) triggers the release of damage-associated molecular patterns (DAMPs), leading to activation of resident macrophages and recruitment of circulating monocytes. Depending on the microenvironment (e.g., IFN-γ/LPS vs. IL-4/IL-13/IL-10/Treg), macrophages polarize into either M1 or M2 phenotypes. M1 macrophages drive acute injury and rejection through pro-inflammatory mediators, while M2 macrophages exhibit a dual role: promoting tissue repair and tolerance (M2a) or, when dysregulated, driving fibrosis (M2c). Chronic inflammation and fibrosis ultimately lead to graft dysfunction, whereas balanced M2-mediated regulation supports long-term graft survival.

Beyond the classical M1/M2 dichotomy, the application of technologies such as single-cell RNA sequencing has revealed the high degree of heterogeneity of ^+^macrophages within the transplant microenvironment. For example, in an allergic airway inflammation model, co-exposure to diesel exhaust particles (DEP) and house dust mite (HDM) can induce the expansion of a unique monocyte-derived macrophage subset (RM.Gp2) characterized as Cd11c^+^, SiglecF^–^, Apoe^+^, Gpnmb^+^, which highly expresses the type 2 chemokines Ccl8 and Ccl24, thereby promoting the recruitment of Th2 cells and eosinophils (12). In leprosy, single-cell sequencing found that Mycobacterium leprae-infected monocyte-derived macrophages mainly exhibit an M2 phenotype with high expression of NEAT1, CCL2, and CD163, and ferroptosis is positively correlated with M2 polarization (2). These studies emphasize the need to move beyond the traditional M1/M2 framework in the transplant context and to define and understand the functions of macrophage subsets from a more refined perspective. Moreover, extracellular vesicles (EVs) released by macrophages and their carried non-coding RNAs (e.g., miRNA, tRNA fragments) also participate in intercellular communication, with M1 and M2 macrophage-derived EVs possessing distinct RNA cargo, which may differentially impact recipient cell function (13). Therefore, in-depth analysis of these heterogeneous subsets and their communication networks will provide a critical basis for developing more precise immune intervention strategies (**Figure 1**).

## 2 The organ transplant microenvironment and the initiation of macrophage recruitment

### 2.1 Ischemia-reperfusion injury as the initial trigger of macrophage activation

In the process of organ transplantation, the unavoidable ischemic period is the initiating event that triggers the subsequent sterile inflammatory cascade. When graft blood flow is interrupted, cells suffer from hypoxia, leading to blocked oxidative phosphorylation, ATP depletion, and ionic pump dysfunction, which in turn cause severe metabolic derangement and cell death (14). Upon reperfusion, the sudden restoration of oxygen not only fails to immediately repair the damage but instead generates large amounts of reactive oxygen species (ROS) through the aberrant activation of mitochondrial respiratory chain complex I, and induces necrotic or apoptotic cells to release a series of endogenous molecules, i.e., DAMPs, such as high mobility group box 1 (HMGB1), ATP, and mitochondrial DNA (15). These DAMPs serve as crucial danger signals, recognized by pattern recognition receptors (such as Toll-like receptors, TLRs, and the NLRP3 inflammasome) on the surface of resident macrophages, thereby rapidly activating them and driving their polarization towards a pro-inflammatory M1-like phenotype (3, 16). For instance, in myocardial IRI, DAMPs drive macrophage M1 polarization via the TLR4/NF-κB signaling pathway, releasing large amounts of pro-inflammatory factors (17), which is consistent with findings in hepatic and renal IRI models, confirming the central role of this signaling axis in sterile inflammation during organ transplantation. Simultaneously, activated vascular endothelial cells upregulate the expression of adhesion molecules (such as selectins, ICAM-1) and secrete chemokines upon DAMP stimulation, with CCL2 (MCP-1) and CX3CL1 being particularly critical (18). These chemokines form a concentration gradient within the graft microenvironment, potently recruiting CCR2-expressing inflammatory monocytes (Ly6C high) from the circulating blood to the injury site (19). Studies have shown that in a lung transplantation model, donor-derived non-classical monocytes, through NLRP3 inflammasome-dependent IL-1β production, activate alveolar macrophages to release CCL2, thereby mediating the recruitment of recipient classical monocytes, a key step leading to primary graft dysfunction (20). Similarly, in liver IRI, antibiotic pretreatment can reshape the gut microbiota and bile acid metabolism, activating the farnesoid X receptor (FXR), which in turn inhibits TLR4 promoter activity and reduces CCL2-CCR2 axis-mediated monocyte recruitment, thus attenuating injury (21). Notably, the chemokine concentration gradient established by early-activated M1 macrophages and endothelial cells is the primary driving force for the rapid expansion of the macrophage pool early after transplantation, as the massive infiltration of circulating CCR2+ inflammatory monocytes rapidly replaces partially depleted resident macrophages (22, 23). This rapid replacement of donor-derived TRMs by recipient-derived MDMs is a critical turning point in the post-transplant immune response. While TRMs are gradually depleted due to IRI and may be only partially replenished, MDMs become the dominant macrophage population within the graft. These recipient-derived MDMs exhibit distinct functional properties compared to their donor-derived counterparts, including heightened responsiveness to inflammatory stimuli and altered metabolic profiles, which collectively shape the trajectory of graft injury, repair, and fibrosis (4). In kidney IRI, the mechanosensitive ion channel Piezo1 upregulates the CCL2/CCR2 pathway via calcium-dependent calpain/HIF-1α signaling, driving M1 macrophage polarization and further exacerbating early inflammatory injury (24). Therefore, IRI, through DAMP release, resident macrophage M1 polarization, and chemokine-mediated massive recruitment of inflammatory monocytes, constitutes the initial trigger of sterile inflammation early after transplantation, laying the critical cellular and molecular foundation for subsequent graft injury, repair, and fibrosis processes.

### 2.2 Allorecognition and adaptive immune signals reprogram macrophages

In organ transplantation, allorecognition is the initial event triggering the adaptive immune response. Recipient antigen-presenting cells (APCs) capture, process, and present donor antigens to T cells, a process central to the activation of adaptive immunity. Activated T cells, particularly Th1 cells, secrete large amounts of IFN-γ. As a potent pro-inflammatory cytokine, IFN-γ can act synergistically with TLR signals on macrophages, strongly driving their polarization towards the classically activated M1 phenotype (25). These M1 macrophages possess potent pro-inflammatory and killing functions, serving as key effector cells in early graft injury and acute rejection. Studies in nanomaterial-induced pulmonary inflammation models have shown that M1 macrophages peak in the early phase (day 1), accompanied by high levels of pro-inflammatory lipid mediators such as leukotriene B4 (LTB4) and prostaglandin E2 (PGE2) (25). In the transplant context, this polarization process directly exacerbates the inflammatory microenvironment within the graft.

Notably, activated macrophages are themselves efficient APCs. Through high surface expression of major histocompatibility complex class II (MHC-II) molecules, they can continuously process and present alloantigens, thereby further amplifying the T cell response (26). This forms a positive feedback loop: T cell-secreted IFN-γ promotes macrophages towards M1 polarization and enhances their antigen-presenting capacity, while activated M1 macrophages in turn activate more T cells, leading to an amplification cascade of the inflammatory response. This positive feedback mechanism is a significant reason why acute rejection is difficult to control. Additionally, in tissue-resident macrophages, metabolic states are closely linked to functional phenotypes; for example, acetyl-CoA carboxylase activity correlates with efferocytosis capacity, and immune perturbations (such as parasitic infection) can induce metabolic reprogramming and alternative activation in monocyte-derived macrophages (27). This indicates that in the post-transplant inflammatory environment, the metabolic adaptability of macrophages also participates in steering their polarization direction.

In the effector phase of adaptive immunity, macrophages act far beyond antigen presentation. In acute cellular rejection (ACR), donor−reactive CD4+ Th1 and Th17 cells are key effectors. They secrete large amounts of IFN−γ and GM−CSF, which potently drive infiltrating monocytes toward the M1 phenotype. In turn, M1 macrophages, through high expression of MHC−II and co−stimulatory molecules and secretion of IL−12 and IL−23, amplify and sustain Th1/Th17 responses, forming a self−reinforcing inflammatory loop. In antibody−mediated rejection (AMR), donor−specific antibodies (DSAs) bind to graft endothelial cells, activating complement and, more importantly, engaging Fcγ receptors on infiltrating macrophages. This engagement activates macrophages, triggering release of TNF−α and IL−1β, and mediates antibody−dependent cell−mediated cytotoxicity (ADCC) against endothelial cells. Thus, the complex immune network driven by HLA mismatches, involving T cells, B cells, and macrophages, collectively dictates the transition from acute injury to chronic fibrosis.

However, in the presence of immunosuppressive therapy or regulatory T cells (Tregs), signals within the microenvironment attempt to shift macrophages towards an M2-like or regulatory phenotype. IL-10 and TGF-β are key anti-inflammatory cytokines in this context. These signals can inhibit M1 polarization and promote the conversion of macrophages to the alternatively activated M2 phenotype (28). M2 macrophages play important roles in tissue repair, fibrosis, and immunoregulation. For example, in a carbon nanotube-induced lung inflammation model, as inflammation transitions from the acute to chronic phase, M2 macrophages peak on day 7, accompanied by high levels of pro-resolving lipid mediators such as resolvin D1 (RvD1) and E1 (RvE1), contributing to the active resolution of inflammation (25). In transplantation, Tregs, by secreting IL-10 and TGF-β, not only directly suppress effector T cell function but also induce macrophages to polarize towards an M2-like phenotype, thereby promoting graft tolerance. This shift from M1 to M2 is crucial for controlling inflammation, promoting graft repair, and preventing chronic rejection. However, excessive or dysregulated M2 polarization can also lead to graft fibrosis. Therefore, the reprogramming of macrophages by adaptive immune signals triggered by allorecognition is a dynamically balanced process, the outcome of which depends on the relative strength of pro-inflammatory and anti-inflammatory signals and profoundly influences graft fate.

## 3 Molecular regulatory networks of macrophage polarization phenotypes

### 3.1 Key signaling pathways: JAK/STAT, NF-κB, and metabolic reprogramming

The regulation of macrophage polarization involves multiple highly conserved and interwoven signaling pathways, among which the JAK/STAT pathway is a central hub determining the direction of M1 versus M2 polarization. Classically activated M1 polarization is primarily triggered by IFN-γ, which, upon binding to its receptor, activates JAK1 and JAK2, subsequently phosphorylating STAT1. Activated STAT1 forms homodimers that translocate to the nucleus and drive the transcription of pro-inflammatory genes such as interferon regulatory factor 8 (IRF8) and inducible nitric oxide synthase (NOS2), endowing macrophages with potent bactericidal and pro-inflammatory capabilities (29). M2 polarization is mainly induced by IL−4 and IL−13. IL−4 signals through the type II IL−4 receptor composed of IL−4Rα and γc, whereas IL−13 primarily engages the type II receptor consisting of IL−4Rα and IL−13Rα1; both activate JAK1 and JAK3, leading to STAT6 phosphorylation. Notably, a type I IL−4 receptor (IL−4Rα/IL−13Rα2) also exists and may mediate more complex functions, offering potential for selective therapeutic targeting (30, 31). STAT6 activation is a critical step for the upregulation of M2 markers such as arginase 1 (Arg1), mannose receptor C-type 1 (Mrc1), and Fizz1 (32). Studies show that polarization direction can be modulated by intervening in the JAK/STAT signaling axis; for example, leflunomide reduces M2 polarization by inhibiting STAT6 phosphorylation, thereby alleviating vascular fibrosis (33), while chitooligosaccharides can simultaneously modulate M1 and M2 polarization by inhibiting both JAK2/STAT1 and JAK1/STAT6 pathways, exerting anti-hepatic fibrosis effects (34).

Besides the JAK/STAT pathway, the Toll-like receptor (TLR)-NF-κB pathway is another key signaling hub driving M1 polarization. During organ transplantation, IRI causes cell death and the release of DAMPs, which, along with pathogen-associated molecular patterns (PAMPs), can be recognized by TLR4 (35). Activation of TLR4, through a MyD88-dependent pathway, ultimately leads to the nuclear translocation of NF-κB, initiating the transcription of a series of pro-inflammatory cytokines (such as TNF-α, IL-6, IL-1β) and chemokines (36). Persistent activation of TLR4/NF-κB signaling is closely associated with chronic inflammation and graft rejection. For instance, in a cisplatin-induced acute kidney injury model, inhibiting the TLR4/NF-κB/MAPK pathway alleviated the inflammatory response (37). Furthermore, the RANKL-RANK signaling system was found to protect renal function after IRI by downregulating TLR4 and MyD88 expression, thereby reducing inflammation (36). This indicates complex crosstalk between signaling pathways mediated by different receptors, collectively shaping the final functional state of macrophages.

The polarization process of macrophages is accompanied by profound metabolic reprogramming, which is not merely a concomitant phenomenon but a driving force for the maintenance and transition of their functional states. M1 polarized macrophages rely on aerobic glycolysis and the pentose phosphate pathway (PPP) to rapidly generate ATP and biosynthetic precursors, meeting the high energy demands for rapid bacterial killing and secretion of large amounts of cytokines. This metabolic pattern, known as the “Warburg effect,” is critically regulated by factors such as mTOR and HIF-1α. Studies have shown that RNA-binding motif protein 4 (RBM4) can negatively regulate M1 polarization by degrading STAT1 mRNA to inhibit glycolysis (29). In contrast, M2 polarized macrophages primarily rely on oxidative phosphorylation (OXPHOS) and fatty acid oxidation (FAO) for energy, a metabolic pattern supporting their roles in tissue repair and anti-inflammatory functions. Lipid metabolism is particularly important in M2 polarization; tumor-associated macrophages (TAMs) drive M2 polarization through enhanced fatty acid oxidation to promote tumor growth (38). This metabolic reprogramming also operates in the transplant microenvironment, indicating that targeting lipid metabolism may be a potential strategy to modulate M2 polarization and combat fibrosis. Additionally, miR-9-5p can protect mitochondrial function and FAO, thereby attenuating renal fibrosis, further confirming the central role of metabolic reprogramming in regulating macrophage function and fibrotic processes (39). Therefore, targeting these key signaling pathways and metabolic checkpoints offers new therapeutic strategies for modulating macrophage polarization and mitigating IRI and fibrosis in organ transplantation (40).

### 3.2 Epigenetic and post-transcriptional regulation

The polarization state of macrophages is precisely regulated by epigenetic modifications and post-transcriptional regulatory mechanisms, which determine the accessibility and expression patterns of polarization-related genes and confer a certain “memory” capacity to macrophages, a factor particularly important in chronic post-transplant inflammatory responses. At the epigenetic level, histone modifications and DNA methylation are key switches controlling macrophage polarization fate. For example, gene loci associated with pro-inflammatory M1 polarization are often accompanied by the enrichment of activating histone modifications such as H3K4me3, enhancing their transcriptional activity (41). Conversely, genes related to anti-inflammatory M2 polarization may be suppressed by repressive modifications like H3K27me3, or activated through specific demethylation processes (42). The dynamic changes in DNA methylation status also profoundly impact macrophage function. Studies indicate that during chronic post-transplant rejection, the methylation profiles of macrophage polarization-related genes may be altered, thereby influencing the maintenance of their long-term inflammatory phenotype (40). These epigenetic marks not only determine the macrophage response to initial stimuli but can also serve as a form of “memory,” affecting their response to subsequent microenvironmental signals (such as persistent post-transplant immune attacks or tissue damage signals), thereby playing a sustained role in chronic pathological processes like graft fibrosis (43).

This ‘memory’ effect mediated by epigenetic modifications is closely related to the concept of ‘trained immunity’. Trained immunity refers to the long−term functional reprogramming of innate immune cells (e.g., monocytes/macrophages) after an initial encounter with a stimulus (such as IRI or LPS), which is driven by persistent epigenetic changes (e.g., H3K4me3 modifications) and metabolic shifts (toward glycolysis). Upon secondary challenge, these cells mount a stronger or more biased response. In the context of transplant IRI, this mechanism is particularly relevant: IRI itself acts as a potent ‘training’ signal, potentially conferring a sustained pro−inflammatory phenotype on graft macrophages, which may then polarize more readily toward M1 upon subsequent alloantigen stimulation, exacerbating rejection (44). Understanding and intervening in this trained immunity process may open new therapeutic windows to prevent the progression from early graft injury to chronic rejection.

At the post-transcriptional regulatory level, non-coding RNAs, especially microRNAs (miRNAs), act as fine-tuners. They regulate the direction of macrophage polarization by targeting the mRNA of key signaling molecules or transcription factors. For instance, miR-155 is widely recognized as a key molecule promoting M1 polarization; it enhances pro-inflammatory signaling pathways like NF-κB by inhibiting negative feedback regulators such as SOCS1 (45). In the context of organ transplantation, the infiltration of M1 macrophages within the graft is associated with the upregulation of miR-155, exacerbating tissue injury and rejection (46). Conversely, some miRNAs, such as miR-124 and members of the let-7c family, have been confirmed to promote macrophage polarization towards the M2 phenotype. They function by targeting and inhibiting pro-inflammatory transcription factors (e.g., STAT1) or promoting anti-inflammatory pathways (e.g., STAT3) (47, 48). This miRNA-mediated regulatory network possesses high spatiotemporal specificity, allowing rapid adjustments based on signals such as cytokines and metabolites in the transplant microenvironment, thus achieving fine-tuned control of macrophage function. For example, in a heart transplantation model, exosomes from mesenchymal stem cells carrying sFgl2 promote M2 polarization by activating the SHP2/STAT3 pathway, a process potentially involving specific miRNAs (48). These post-transcriptional regulatory mechanisms intertwine with epigenetic changes, collectively shaping the dynamic plasticity of macrophages within the complex post-transplant immune environment, providing a theoretical basis for developing novel therapeutic strategies targeting macrophage polarization, such as miRNA-based therapies or epigenetic drugs (40).

## 4 The role of M1 polarization in acute graft injury and rejection

### 4.1 Mediating early ischemia-reperfusion injury

In the early stages of organ transplantation, IRI is a key factor leading to graft dysfunction and primary nonfunction, with macrophages, especially early infiltrating M1-type macrophages, playing a core driving role. Liver IRI is a common clinical challenge after liver transplantation and partial hepatectomy, involving complex sterile inflammatory responses (35). Studies indicate that macrophage-mediated excessive inflammatory responses are a significant factor in liver IRI, where M1 macrophages exacerbate the injury by promoting inflammatory progression (3). These activated M1 macrophages produce large amounts of reactive oxygen species (ROS) and reactive nitrogen species (RNS), directly causing oxidative stress damage to endothelial and parenchymal cells. For example, during liver transplant IRI, reperfusion-triggered ROS production is a critical step leading to hepatocyte injury and eventual organ failure (35). Furthermore, in fatty liver models, the S100A9 protein induces M1 macrophage polarization via the TLR4/NF-κB signaling axis, thus exacerbating hepatic inflammatory injury (17). This oxidative stress not only directly destroys cellular structures but also increases vascular permeability, creating conditions for subsequent inflammatory cell infiltration.

Pro-inflammatory cytokines secreted by M1 macrophages, such as TNF-α and IL-1β, are crucial mediators that induce parenchymal cell apoptosis and necroptosis and activate the complement system, forming a vicious inflammatory cycle. In a renal IRI model, the receptor activator of NF-κB ligand (RANKL) system improved renal function by influencing macrophage function and downregulating TLR4, thereby reducing TNF-α and IL-6 production, inversely confirming the central role of these factors in injury (36). Similarly, in liver transplant IRI, the activation of the NF-κB signaling pathway is closely associated with the release of pro-inflammatory factors (17). These cytokines not only directly induce parenchymal cell death but also further activate local innate and adaptive immune responses, forming a self-amplifying inflammatory loop. For instance, in cardiac IRI, M1 macrophage-mediated pyroptosis has been shown to exacerbate myocardial injury (49). Pyroptosis, as a highly inflammatory form of programmed cell death, significantly aggravates liver IRI when its executive protein GSDMD is activated in innate immune cells like macrophages (50). The persistence of this inflammatory cycle severely impedes early graft recovery.

Furthermore, activated macrophages may degrade the extracellular matrix (ECM) and basement membrane by releasing matrix metalloproteinases (MMPs) and other proteolytic enzymes, disrupting tissue structure integrity and paving the way for further inflammatory cell infiltration and tissue remodeling. While the specific role of macrophage-released MMPs in transplant IRI was not directly detailed in the provided references, the imbalance in macrophage polarization itself is central to tissue destruction and inflammatory spread. For example, during the transition from renal IRI to chronic kidney disease, the sustained activation and infiltration of macrophages are important factors leading to tubulointerstitial fibrosis (51). Abnormal regulation of macrophage polarization, such as the loss of tuberous sclerosis complex 1 (TSC1), promotes fibrosis during the repair phase following IRI, indirectly reflecting the macrophage-mediated tissue remodeling process (52). The acidic microenvironment has also been shown to promote macrophage polarization towards the M1 phenotype by regulating peroxisome proliferator-activated receptor γ (PPARγ) signaling, thereby aggravating the severity of liver IRI (53). In summary, early infiltrating M1 macrophages play a pivotal role in the initiation and amplification of early organ transplant IRI through multiple mechanisms, including the production of ROS/RNS, release of pro-inflammatory cytokines, and potential participation in matrix degradation. Intervention targeting their polarization process has become an important strategic direction for improving transplant prognosis.

### 4.2 Driving acute cellular rejection

In acute cellular rejection (ACR), M1 macrophages serve as key effector cells, with their function significantly amplified by T cell-derived IFN-γ signals. IFN-γ is a potent inducer of M1 polarization, driving macrophages to differentiate into the pro-inflammatory M1 phenotype by activating the JAK/STAT1 signaling pathway (54). Polarized M1 macrophages highly express inducible nitric oxide synthase (iNOS) and various pro-inflammatory cytokines such as TNF-α, IL-6, and IL-1β (46). These cytokines and mediators can directly damage graft cells expressing alloantigens. Moreover, M1 macrophages can directly kill target cells through antibody-dependent cell-mediated cytotoxicity (ADCC). In antibody-mediated rejection (AMR), the Fc portion of donor-specific antibodies (DSA) binds to Fcγ receptors (such as FcγRIIIa/CD16a) on the surface of macrophages, activating them and mediating ADCC, leading to graft vascular endothelial injury (55). Studies have shown significant infiltration of M1 macrophages (with elevated markers CD86+, CD80+) in grafts of patients with renal transplant rejection, while M2 macrophage markers (CD163+, CD206+) are decreased, suggesting that M1/M2 imbalance is an important feature of rejection (46).

Another core role of M1 macrophages in ACR is acting as APCs and immunoregulatory cells, maintaining and amplifying Th1 and Th17 immune responses. M1 macrophages present alloantigen peptides via MHC class II molecules to activate naive CD4+ T cells. Simultaneously, they secrete IL-12, a key cytokine driving Th1 differentiation, and IL-23, which is crucial for the survival and expansion of Th17 cells (56). These cytokines work together to promote the proliferation and function of effector T cells, thereby exacerbating graft injury. Conversely, M1 macrophages inhibit the induction and function of Tregs by secreting pro-inflammatory factors and inhibitory signals. Tregs are key cells for maintaining immune tolerance, and their functional suppression further disrupts the immune balance and promotes rejection. Research confirms that in a skin transplantation model, promoting M2 macrophage polarization and Treg expansion can effectively inhibit effector T cell proliferation and prolong graft survival (57). Additionally, in a heart transplant model, mesenchymal stem cell-derived exosomes carrying soluble fibrinogen-like protein 2 (sFgl2) significantly alleviated acute rejection by promoting M2 polarization, inhibiting M1 polarization, and increasing the proportion of Tregs (48).

**Figure 2:**
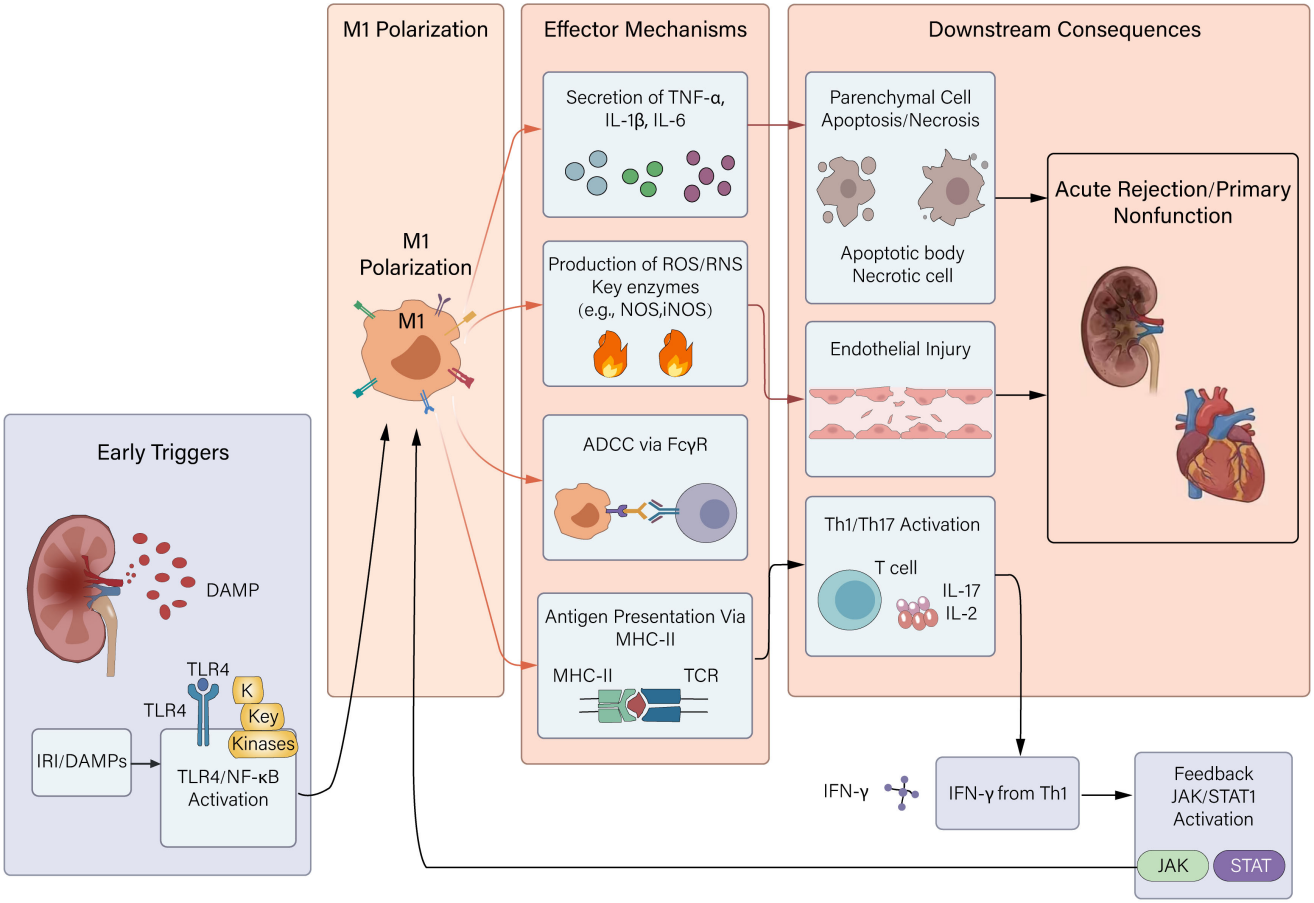
M1 macrophages as key effectors of acute graft injury and rejection. Early triggers such as IRI/DAMPs (via TLR4/NF-κB) and Th1-derived IFN-γ (via JAK/STAT1) drive M1 polarization. M1 macrophages then promote acute rejection through multiple effector mechanisms: (1) secretion of pro-inflammatory cytokines (TNF-α, IL-1β, IL-6) inducing parenchymal cell death; (2) production of reactive oxygen/nitrogen species (ROS/RNS); (3) antigen presentation via MHC-II, amplifying Th1/Th17 responses; and (4) antibody-dependent cell-mediated cytotoxicity (ADCC) via Fcγ receptors, causing endothelial injury. These pathways collectively lead to acute rejection or primary graft nonfunction.

In AMR, M1 macrophages recognize and bind to DSA deposited on the graft vascular endothelium through Fcγ receptors, a critical step leading to endotheliitis and vascular injury. DSA binding to endothelial cells not only activates the complement system, but its Fc portion is also recognized by FcγR on infiltrating macrophages, thereby activating them (55). Activated macrophages release pro-inflammatory cytokines like TNF-α and IFN-γ, which can induce phenotypic changes in endothelial cells, promoting coagulation, inflammation, and increased vascular permeability (55). Furthermore, M1 macrophages can directly kill endothelial cells through ADCC. Studies have shown that in a renal transplant AMR model, M1 macrophage polarization exacerbated rejection by promoting DSA-mediated endothelial cell injury (58). Further research has found that endothelial cells activated by human leukocyte antigen (HLA) class I antibodies can induce monocyte polarization towards M2-type macrophages via TLR4 signaling and P-selectin, leading to the secretion of matrix metalloproteinase 9 (MMP9), which participates in vascular remodeling and the pathogenesis of AMR (59). This suggests that macrophage function in AMR is complex, with M1 and M2 subsets potentially playing synergistic or antagonistic roles at different stages or in different microenvironments. In summary, M1 macrophages play a central role in driving both acute cellular and antibody-mediated rejection through direct cytotoxicity, antigen presentation, cytokine secretion, and Fc receptor-mediated effector mechanisms (**Figure 2**).

## 5 The dual role of M2 polarization in graft repair and tolerance induction

M2 polarization is not a single state but a spectrum comprising multiple functional subtypes. In organ transplantation, different M2 subtypes exert divergent and even opposing effects. For example, M2a, induced by IL−4/IL−13, primarily participates in Th2 responses and tissue repair, whereas M2c, driven by IL−10/TGF−β, plays a central role in immunosuppression and fibrosis. Therefore, the following sections will discuss the dual roles of specific M2 subpopulations in graft repair, tolerance induction, and chronic fibrosis, based on their functional contexts.

### 5.1 Promoting tissue repair and regeneration

In the late phase of injury following organ transplantation, or under the regulation of immunosuppressive drugs, M2 macrophages, particularly the M2a subtype, play a central role in suppressing excessive inflammatory responses by secreting anti-inflammatory cytokines such as IL-10 and TGF-β, thereby creating a favorable microenvironment for tissue repair (60, 61). This transition from pro-inflammatory to anti-inflammatory states is key for inflammation resolution and tissue regeneration. Studies indicate that M2 macrophages, through the secretion of IL-10 and TGF-β, effectively inhibit the production of pro-inflammatory cytokines and promote the development of Tregs, thus establishing an immunosuppressive environment at the injured site and preventing excessive immune-pathological damage (62, 63). For example, in an acute liver injury model, adoptively transferred alternatively activated macrophages (AAMs) significantly reduced hepatocyte necrosis and neutrophil infiltration, and lowered the levels of multiple pro-inflammatory cytokines in the circulation, a process closely related to the potent phagocytic activity of AAMs and their interaction with the host innate immune system (64, 65). Moreover, in a diabetic wound model, M2 macrophages reverse the M1-dominated inflammatory state and promote wound healing by secreting factors like IL-10 (66).

In addition to suppressing inflammation, M2 macrophages directly promote angiogenesis and endothelial repair by producing key growth factors such as vascular endothelial growth factor (VEGF) and platelet-derived growth factor (PDGF) (67, 68). VEGF is a primary driver of angiogenesis; M2 macrophage-secreted VEGF stimulates endothelial cell proliferation, migration, and tubular structure formation, providing oxygen and nutrients to the damaged tissue, which is crucial for graft survival and functional recovery (69, 70). PDGF mainly acts on pericytes and vascular smooth muscle cells, promoting the maturation and stabilization of newly formed vessels (71). Studies have shown that in diabetic wounds, M2 macrophages significantly promote neovascularization and improve local blood supply by secreting VEGF and PDGF, thereby accelerating wound closure (72, 73). Furthermore, during bone regeneration, M2 macrophage-derived VEGF and bone morphogenetic protein-2 (BMP-2) synergistically promote not only angiogenesis but also directly induce osteogenic differentiation, achieving the coupling of vascularization and bone formation (74).

M2 macrophages also mediate the clearance of apoptotic cells (efferocytosis) by secreting molecules such as milk fat globule-epidermal growth factor 8 (MFG-E8), a critical step in inflammation resolution and tissue repair (75). Timely clearance of apoptotic cells prevents their secondary necrosis and the release of pro-inflammatory contents, thereby terminating the inflammatory cascade. MFG-E8 acts as a bridging molecule, connecting phosphatidylserine (PS) on the surface of apoptotic cells to integrins on phagocytes, facilitating macrophage recognition and engulfment of apoptotic cells (76). Studies show that M2 macrophages efficiently clear apoptotic cells through the Mer receptor tyrosine kinase (MerTK) and its ligand protein S (ProS1) signaling pathway. This process not only suppresses inflammation but also triggers the production of anti-inflammatory and pro-repair factors such as TGF-β and IL-10, forming a positive feedback loop that drives tissue transition from inflammation to the repair phase (77, 78). In a myocardial infarction model, M2 macrophages, identified by specific imaging of markers like CD206 (mannose receptor), were associated with favorable wound healing and cardiac remodeling, further confirming their critical role in tissue repair (79). Therefore, M2 macrophages, through the triple mechanisms of inhibiting inflammation, promoting angiogenesis, and clearing apoptotic cells, work synergistically to provide the necessary cellular and molecular basis for tissue repair and regeneration after organ transplantation.

### 5.2 Participating in immunoregulation and tolerance

Certain M2 subtypes, particularly those with regulatory functions (such as Mregs), play a key role in inducing and maintaining immune tolerance. These cells exert immunosuppressive effects through multiple mechanisms, for example, they highly express programmed death-ligand 1 (PD-L1) and directly inhibit the proliferation and function of effector T cells by secreting anti-inflammatory factors like IL-10 and TGF-β, while simultaneously promoting the induction and expansion of Tregs (80, 81). Importantly, among these regulatory subsets, regulatory macrophages (Mregs) represent a distinct activation state, typically generated *in vitro* by stimulating monocytes with IFN−γ plus a TLR agonist (e.g., LPS). Their phenotype and function differ significantly from classical M2 macrophages. Mregs suppress effector T−cell proliferation and function by secreting IL−10 and TGF−β, and by highly expressing immunomodulatory molecules such as IDO and iNOS. Concurrently, they promote the induction and expansion of Tregs. In transplant tolerance models, adoptive transfer of donor−antigen−primed Mregs has been shown to prolong graft survival effectively, highlighting their therapeutic potential distinct from that of M2 cells (82). In the tumor microenvironment, this immunosuppressive state dominated by M2-like macrophages is closely associated with resistance to immune checkpoint inhibitors like anti-PD-L1 therapy (80). For instance, in hepatocellular carcinoma, downregulation of the angiotensin-converting enzyme 2 (ACE2) axis leads to an increase in M2-like tumor-associated macrophages, which exhibit a CCR5+PD-L1+ immunosuppressive phenotype and secrete high levels of VEGFα, collectively constituting resistance to PD-L1 blockade therapy (80). Furthermore, in bladder cancer, the CD39/CD73-mediated adenosinergic pathway is closely associated with an increase in M2-like macrophages and a decrease in effector T cells, further reinforcing the local immune-tolerant environment (81). These mechanisms indicate that specific M2-like macrophage subsets are core executors in shaping the immunosuppressive microenvironment and causing immunotherapy failure.

In models of operational tolerance or under certain successful immunosuppressive regimens, a predominant infiltration of M2-like macrophages is often observed within the graft, suggesting a strong correlation with long-term graft survival and the establishment of a tolerant state. For example, during successful embryo implantation, the accumulation of CD206+ M2-like macrophages in the endometrium is crucial for establishing maternal-fetal immune tolerance, and their absence leads to implantation failure (83). Similarly, in studies of recurrent pregnancy loss, conditioned medium from chorionic villus trophoblasts could induce tolerogenic M2-like macrophages and expand Treg cells, a capacity maintained in individuals with successful pregnancies, indicating a fundamental role for M2-like macrophages in maintaining pregnancy tolerance (84). In the context of solid organ transplantation, M2 macrophages are considered key innate immune executors in chronic rejection, but they may also participate in promoting immune tolerance under specific conditions (85). In oncology, successful therapeutic interventions can sometimes remodel macrophage phenotypes; for instance, in a glioblastoma model, a ketogenic diet, despite its anti-tumor activity, unexpectedly increased the proportion of immunosuppressive M2-like macrophages, and combination therapy with a colony-stimulating factor 1 receptor inhibitor (BLZ945) to correct this polarization significantly improved survival, inversely confirming the correlation between M2-like macrophage predominance and immune-tolerant states (86).

However, this regulatory function of macrophages is not static; their phenotype possesses high plasticity and can undergo conversion when microenvironmental signals change, leading to instability in the immunoregulatory state. Macrophage polarization is precisely regulated by multiple signaling pathways, including JAK-STAT, PI3K-Akt, and PPARγ (87, 88). For example, long-term hyperglycemic environments can disrupt the typical functional dichotomy of M1 and M2 macrophages, causing an M2-like to M1-like phenotypic shift with impaired repair capacity, revealing how metabolic stress can disrupt established immunoregulatory programs (89). In an inflammatory environment, ectosomes carrying the ion channel Calhm6 can promote M2-like polarization and induce immune tolerance, while Calhm6 deficiency enhances M1-like polarization (90). Additionally, tumor-derived signaling molecules such as secreted phosphoprotein 1 (SPP1) can recruit and polarize APOE+ M2-like macrophages to the tumor margin; these cells shape the immunosuppressive microenvironment by releasing signals like TGF-β, but this polarized state can be affected by targeted interventions (91). Similarly, in non-small cell lung cancer, the expression of M2-like macrophage-related genes is upregulated around persistent cancer cells left after treatment with epidermal growth factor receptor tyrosine kinase inhibitors, promoting immune tolerance; combining CSF-1R inhibitor therapy to deplete these macrophages can enhance treatment efficacy (92). Collectively, these studies indicate that although M2-like macrophages contribute to immunoregulation at specific stages, their phenotype and function are highly susceptible to local cytokines, metabolites, mechanical forces, and therapeutic interventions. This instability is a core challenge that must be considered when designing tolerance induction strategies based on macrophage polarization modulation.

## 6 Macrophage polarization and the development of chronic graft fibrosis

Traditionally, M2 macrophages, particularly the M2a subtype, are considered core drivers of fibrosis. However, recent single−cell studies have identified pro−fibrotic macrophage subsets (e.g., SPP1+, TREM2+, scar−associated macrophages) whose transcriptomic profiles do not fully align with the classical M2 definition (10.1126/sciimmunol.add8945). Nevertheless, these subsets still highly express multiple pro−fibrotic factors, and their functions largely overlap with M2a macrophages. In this review, we use the term ‘M2/M2−like’ to denote pro−fibrotic macrophage populations, while acknowledging the limitations of this nomenclature. We further discuss this complexity and the need to move beyond the M1/M2 dichotomy in Section 7.1.

### 6.1 M2 polarization is a core driver of the fibrotic process

After organ transplantation, long-term allogeneic immune responses, combined with non-immune factors (such as hypertension, hyperlipidemia, and drug toxicity), collectively create a microenvironment of persistent low-grade inflammation and injury. This microenvironment is a key factor driving macrophages towards a pro-fibrotic M2-like phenotype, particularly the M2a subtype (93). M2a macrophages are considered an important cellular source of TGF-β, the most potent cytokine for activating fibroblast differentiation into myofibroblasts, thereby stimulating excessive extracellular matrix (ECM) synthesis. However, it should be noted that myofibroblasts themselves, as well as regulatory T cells (Tregs) and other stromal cells, also contribute substantially to TGF-β production within the fibrotic microenvironment, and the relative contribution of each source may vary depending on the stage and context of fibrosis (94). Studies have shown that in disease models such as chronic pancreatitis and pulmonary fibrosis, M2 macrophage infiltration positively correlates with TGF-β1 expression levels, and TGF-β1 is mainly produced by CD206^+^ M2 macrophages (94, 95), suggesting that this M2−TGF−β1 pro−fibrotic axis is conserved across tissues and represents a core mechanism driving chronic graft fibrosis after transplantation. Furthermore, M2 macrophages can directly stimulate fibroblast proliferation and collagen deposition by secreting PDGF and connective tissue growth factor (CTGF) (96). For example, in a spinal cord injury model, M2 macrophage-secreted PDGFB acts on PDGFRβ^+^ pericytes, promoting their migration to the injury core and participating in fibrotic scar formation (97). Meanwhile, CTGF, as a key pro-fibrotic factor secreted by M2 macrophages, mediates fibroblast proliferation and migration by activating the AKT, ERK1/2, and STAT3 signaling pathways, playing a core role in wound healing and organ fibrosis (96). In renal fibrosis, succinate, through its receptor SUCNR1, activates the p-Akt/p-GSK3β/β-catenin signaling pathway, promoting CTGF transcription in M2 macrophages and thereby mediating the crosstalk between macrophages and fibroblasts, exacerbating renal interstitial fibrosis (98). Additionally, in a chronic obstructive nephropathy model, NLRX1 deficiency promotes M2 macrophage polarization and enhances TGF-β secretion, aggravating renal tubular injury and fibrosis (99). This evidence indicates that M2 polarization, especially the M2a subtype, constitutes a core driving mechanism of organ fibrosis through the secretion of multiple pro-fibrotic factors such as TGF-β, PDGF, and CTGF.

### 6.2 Macrophage-myofibroblast crosstalk

Complex paracrine dialogue exists between macrophages and activated myofibroblasts, and this bidirectional signaling communication is a core driving force of fibrosis progression after organ transplantation, ultimately forming a pro-fibrotic positive feedback loop. Activated myofibroblasts are one of the main sources of monocyte chemoattractant protein-1 (MCP-1/CCL2). This chemokine, by binding to its receptor CCR2, continuously recruits peripheral blood monocytes to the injury site and promotes their differentiation into macrophages (100). In a myocardial IRI model, CD11b blockade significantly reduced neutrophil and monocyte infiltration and inhibited fibroblast activation, thus alleviating myocardial fibrosis (101). Similarly, in diabetic nephropathy, a high-glucose environment induces senescence in renal tubular epithelial cells, which secrete Shh, in turn activating fibroblasts and upregulating MCP-1 expression, exacerbating the inflammatory response and fibrotic process (102). Furthermore, in peri-implant tissues from metal-on-metal total hip arthroplasty revisions, activated synovial fibroblasts promote immune cell infiltration and monocyte differentiation by secreting MCP-1, leading to excessive local macrophage accumulation (103). These recruited macrophages, particularly M2-type macrophages, further maintain and enhance myofibroblast activation by secreting cytokines such as TGF-β and IL-10, thereby forming a self-reinforcing vicious cycle (104, 105).

**Figure 3:**
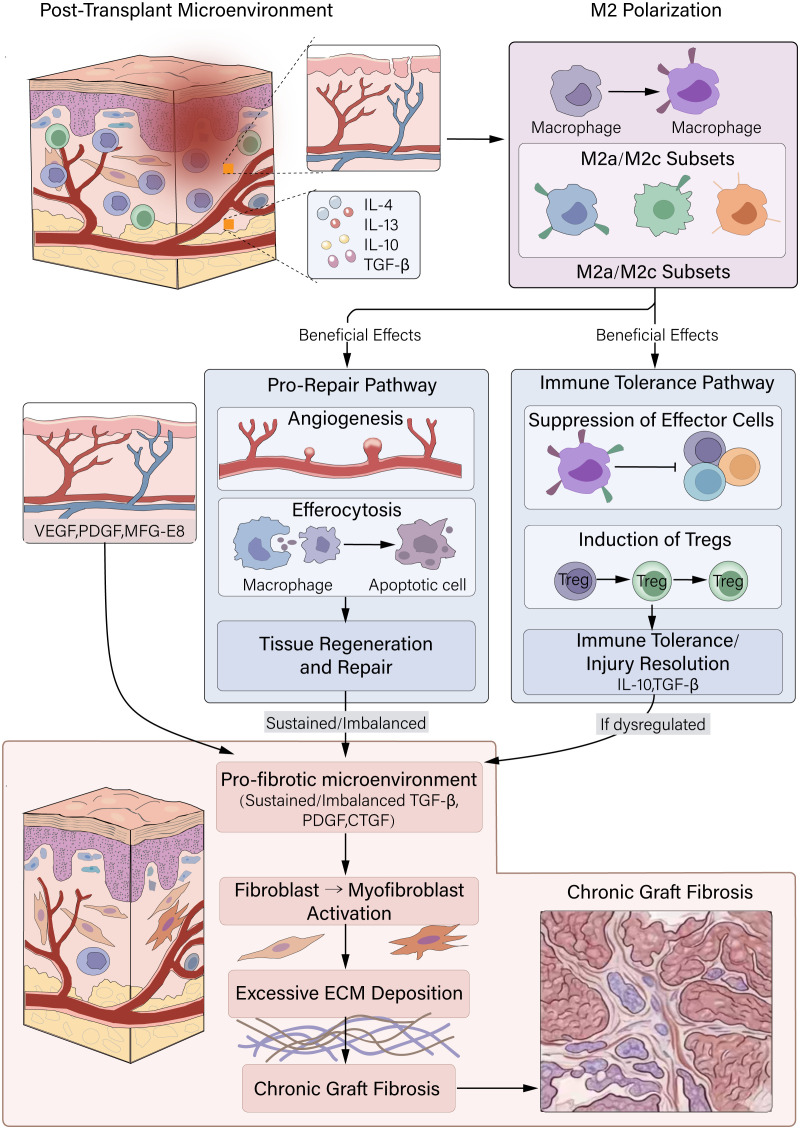
The paradoxical roles of M2 macrophages in graft repair and fibrosis. Under the influence of IL-4, IL-13, IL-10 and TGF-β, macrophages polarize to M2 subsets (M2a, M2c). On one hand, M2 macrophages promote beneficial outcomes: they secrete IL-10 and TGF-β to suppress effector T cells and induce Tregs, facilitating immune tolerance and injury resolution; they also produce VEGF, PDGF, and MFG-E8 to support angiogenesis and efferocytosis, driving tissue repair. On the other hand, persistent or dysregulated M2 activation leads to excessive secretion of pro-fibrotic factors (TGF-β, PDGF, CTGF), which convert fibroblasts to myofibroblasts and cause pathological extracellular matrix (ECM) deposition, ultimately resulting in chronic graft fibrosis.

Macrophages also finely regulate the balance between ECM degradation and deposition by secreting MMPs and their tissue inhibitors (TIMPs). In the early stages of fibrosis, macrophage-secreted MMPs help clear damaged ECM, creating conditions for tissue repair. However, in the middle and late stages of fibrosis, the macrophage phenotype shifts towards a pro-fibrotic direction, leading to the overexpression of TIMPs, which inhibits MMP activity, resulting in insufficient ECM degradation and ultimately irreversible fibrosis. Studies indicate that in idiopathic pulmonary fibrosis, activation of TAM receptor signaling (Tyro3, Axl, Mer) is closely associated with fibroblast proliferation, migration, and pro-fibrotic gene expression, and inhibiting this pathway can modulate macrophage polarization and influence ECM remodeling (106). In acute kidney injury post-renal transplantation, single-nucleus RNA sequencing and imaging mass cytometry revealed complex interactions among epithelial cells, immune cells, and stromal cells, where differences in macrophage polarization direction determine whether the injury proceeds towards repair or fibrosis; non-recovering grafts were enriched with pro-fibrotic macrophages and dendritic cells, spatially colocalized with activated fibroblasts in fibrotic areas (107). Additionally, after myocardial IRI, an S100a9 high-expressing macrophage subset was identified as a key cell linking acute inflammation to fibrotic remodeling. These cells not only amplify the inflammatory response by activating the Myd88/NFκB/NLRP3 signaling pathway but also induce fibroblast-to-myofibroblast transformation and macrophage-to-myofibroblast transition (MMT) via the TGF-β/p-smad3 signaling pathway, thus promoting fibrosis progression (108). Therefore, the crosstalk between macrophages and myofibroblasts, along with macrophage-mediated regulation of the ECM degradation/deposition balance, collectively determines the outcome of fibrosis after organ transplantation (**Table 1**). Targeting this interactive network holds promise as a novel strategy for anti-fibrotic therapy (**Figure 3**).

**Table 1:** Key macrophage subsets in transplantation.

Subset	Inducing signals	Surface markers	Key effector molecules	Functional role in transplantation	Therapeutic implications
M1	IFN-γ, LPS, DAMPs	CD86, CD80, iNOS	TNF-α, IL-1β, IL-6, ROS, NO	Pro-inflammatory; drives IRI and acute rejection	Inhibit STAT1/NF-κB to reduce early injury
M2a	IL-4, IL-13	CD206, Arg1	TGF-β, IL-10, PDGF, VEGF	Tissue repair, angiogenesis; but may promote fibrosis if sustained	PPAR-γ/STAT6 agonists with caution for pro-fibrotic risk
M2b	Immune complexes + TLR ligands	CD86, IL-1R	IL-10, IL-12, TNF-α	Immunomodulatory; may contribute to chronic inflammation	Targeting FcγR signaling
M2c	IL-10, TGF-β, glucocorticoids	CD163, MerTK	IL-10, TGF-β, MMPs	Immunosuppression, efferocytosis; strong pro-fibrotic potential	Avoid over-activation; block TGF-β/CTGF
Mregs	IFN-γ + TLR agonist (in vitro)	IDO, iNOS, IL-10	IL-10, TGF-β, IDO, iNOS	Potent T-cell suppression; promote tolerance	Adoptive transfer of donor-specific Mregs for tolerance induction

## 7 Therapeutic strategies targeting macrophage polarization and research progress

### 7.1 Pharmacological intervention: modulating polarization signaling pathways

Pharmacological intervention targeting macrophage polarization signaling pathways is an important strategy for regulating immune responses in organ transplantation. Developing small molecule inhibitors targeting key pathways of M1 polarization, such as STAT1 and NF-κB, has shown potential in animal models to alleviate early inflammation and acute rejection. Research indicates that M1 macrophages exacerbate graft injury by secreting pro-inflammatory factors like TNF-α and IFN-γ, and inhibiting STAT1 signaling can effectively block this process (29). For example, RBM4 protein regulates M1 polarization by targeting STAT1-mediated glycolysis, suggesting STAT1 is a critical node for M1 intervention (29). Moreover, the NF-κB pathway is equally vital in M1 polarization, and its inhibitors can reduce the production of pro-inflammatory factors, thereby mitigating acute rejection (109). In a corneal transplantation model, kaempferol alleviated graft rejection by inducing autophagy to inhibit NLRP3 inflammasome activation and M1 polarization (110). These findings indicate that small molecule inhibitors targeting STAT1 and NF-κB are feasible strategies for attenuating early inflammation and acute rejection.

On the other hand, using drugs such as PPAR-γ agonists (e.g., pioglitazone) and STAT6 agonists to promote M2 polarization can enhance repair and immunoregulatory functions, but caution is needed regarding their potential pro-fibrotic risks. M2 macrophages promote immune tolerance by secreting anti-inflammatory factors like IL-10 and inducing Tregs (57, 111). For instance, ECDI-fixed donor splenocytes induced M2 polarization and Treg generation by promoting CREB phosphorylation, thereby prolonging skin graft survival (57). However, M2 macrophages can also play a negative role in chronic rejection by promoting fibrosis (85). Studies have shown that M2 macrophages are associated with endothelial-to-mesenchymal transition (EndMT) in chronic graft dysfunction, potentially exacerbating fibrotic progression (43). Therefore, when applying PPAR-γ or STAT6 agonists, it is necessary to balance their anti-inflammatory and pro-fibrotic dual effects and explore combination drug strategies to mitigate risks.

Interventions targeting metabolic reprogramming, such as using metformin to enhance oxidative phosphorylation, may help shift macrophages towards a reparative phenotype. The metabolic state of macrophages is closely linked to their polarization direction: M1 macrophages rely on glycolysis, while M2 macrophages are more dependent on oxidative phosphorylation (29). Studies show that metformin, by activating the AMPK pathway to promote oxidative phosphorylation, inhibits M1 polarization and promotes M2 polarization (109). Furthermore, dietary carboxymethylcellulose, through the induction of LPA production, upregulates macrophage glycolysis and promotes M1 polarization, whereas the use of glycolytic inhibitors prolongs graft survival. These findings underscore the importance of metabolic intervention in regulating macrophage polarization (112).By targeting metabolic reprogramming, such as using metformin or glycolytic inhibitors, it is hoped that macrophages can be shifted from a pro-inflammatory phenotype to a reparative one, thereby improving graft outcomes. However, the clinical translation of these strategies still requires further validation of their safety and efficacy.

### 7.2 Cell therapy and genetic engineering

Infusion of ex vivo-induced regulatory macrophages (Mregs) or M2-type macrophages has shown potential in preclinical studies to prolong graft survival and attenuate fibrosis. M2 macrophages play a central role in inducing immune tolerance by secreting anti-inflammatory cytokines (such as IL-10 and TGF-β) and inhibiting effector T cell activation (111). For example, in a skin transplantation model, infusion of ECDI-fixed donor splenocytes promoted recipient macrophage polarization towards the M2 phenotype, accompanied by the expansion of Tregs, significantly prolonging graft survival (57). Similarly, in a renal transplant model of acute T cell-mediated rejection (TCMR), overexpression of IL-34 alleviated rejection by upregulating M2 macrophage polarization, suggesting that IL-34 could be a potential therapeutic strategy (113). Moreover, Schistosoma japonicum-derived cystatin (rSj-Cys) effectively induces macrophage polarization towards the M2 phenotype, and adoptive transfer of these cells significantly improved survival and reduced multi-organ injury in septic mice, a mechanism associated with inhibiting the TLR2/MyD88 signaling pathway (114, 115). In heart transplantation, ultrasound-responsive macrophage-biomimetic phase-change nanoparticles delivering miR-126 not only inhibited the infiltration of macrophages and CD3+ T cells but also promoted M2 macrophage differentiation, thereby attenuating vascular endothelial and interstitial fibrosis and improving microcirculation and cardiac function (116). Collectively, these studies demonstrate that by ex vivo induction or *in vivo* regulation of M2 macrophages, the graft immune microenvironment can be effectively remodeled, providing robust preclinical evidence for prolonging graft survival and inhibiting fibrosis.

Using nanoparticles or adeno-associated viral vectors to deliver polarization-regulating genes (such as IL-10 overexpression) or siRNA/miRNA (such as miR-155 knockdown) to macrophages enables local and specific phenotypic reprogramming, allowing for precise regulation of transplant rejection. Genetic engineering approaches can overcome the systemic side effects of traditional drug therapies and enhance treatment specificity. For example, adeno-associated virus-mediated overexpression of IL-34 in kidney transplant recipients significantly increased the proportion of M2 macrophages and Tregs in the graft and spleen, while downregulating serum levels of pro-inflammatory factors like IFN-γ, IL-17, and TNF-α, thereby effectively reducing the severity of TCMR (113). In heart transplantation, researchers developed miR-126-loaded macrophage-biomimetic phase-change nanoparticles (miR-126-MCNPs) and utilized ultrasound-targeted microbubble destruction for precise delivery, which not only promoted M2 macrophage differentiation but also significantly inhibited vascular endothelial and interstitial fibrosis and promoted neovascularization (116). Furthermore, engineering M2 macrophage-derived exosomes is an emerging strategy. For instance, fibroblast growth factor 21 (FGF21) loaded into M2 macrophage-derived exosomes (FGF21-M2-Exos) enabled sustained release of FGF21 in a sepsis-induced acute lung injury model, alleviating pulmonary inflammation and apoptosis by promoting M2 macrophage polarization and inhibiting glycolysis (117). These studies confirm that reprogramming macrophages via genetic engineering achieves precise control over their polarization state, exerting potent local anti-inflammatory and pro-repair effects, thus opening new avenues for immunotherapy following organ transplantation.

Beyond modulating polarization, enhancing macrophage phagocytic function represents another promising therapeutic strategy. The CD47−SIRPα axis is a critical ‘don’t eat me’ checkpoint. In oncology, tumor cells overexpress CD47 to evade phagocytosis. In transplantation, donor graft cells may similarly upregulate CD47 to resist recipient macrophage clearance. Blocking this pathway with anti−CD47 antibodies or SIRPα fusion proteins can enhance macrophage−mediated clearance of senescent or damaged cells, potentially alleviating chronic inflammation and promoting tissue remodeling. Future studies could explore combining CD47−SIRPα blockade with M2−promoting strategies to simultaneously suppress rejection and facilitate repair (118, 119).

### 7.3 Improving the transplant microenvironment to indirectly regulate polarization

Optimizing immunosuppressive regimens is a key strategy for improving the transplant microenvironment and indirectly regulating macrophage polarization. Traditional calcineurin inhibitors (CNIs), such as tacrolimus and cyclosporine A, can effectively prevent acute rejection, but their side effects, such as nephrotoxicity, are non-negligible. Studies suggest that CNIs may promote aberrant macrophage activation towards a pro-inflammatory (M1) phenotype through pathways like inducing endoplasmic reticulum (ER) stress, thereby exacerbating graft injury (120). ER stress-related proteins such as GRP94 have been identified as novel regulators of M1 macrophage polarization, and their deficiency can suppress M1 polarization and improve insulin resistance (120). Therefore, developing or adopting immunosuppressive regimens that reduce CNI exposure, such as combining mTOR inhibitors or co-stimulation blockers, may indirectly suppress M1 polarization and promote M2 polarization by reducing ER stress, thereby improving long-term graft survival. Additionally, targeted therapies against specific immune cell subsets show promise. For instance, mesenchymal stem cell-derived exosomes (MSC-Exos) carrying sFgl2 can effectively promote M2 macrophage polarization while inhibiting M1 polarization by binding to the CD32b receptor on macrophages and activating the downstream SHP2-STAT3 signaling pathway, significantly alleviating acute rejection in a mouse heart transplant model (48). This strategy provides a new idea for optimizing immunosuppressive regimens by directly modulating macrophage polarization direction.

**Figure 4:**
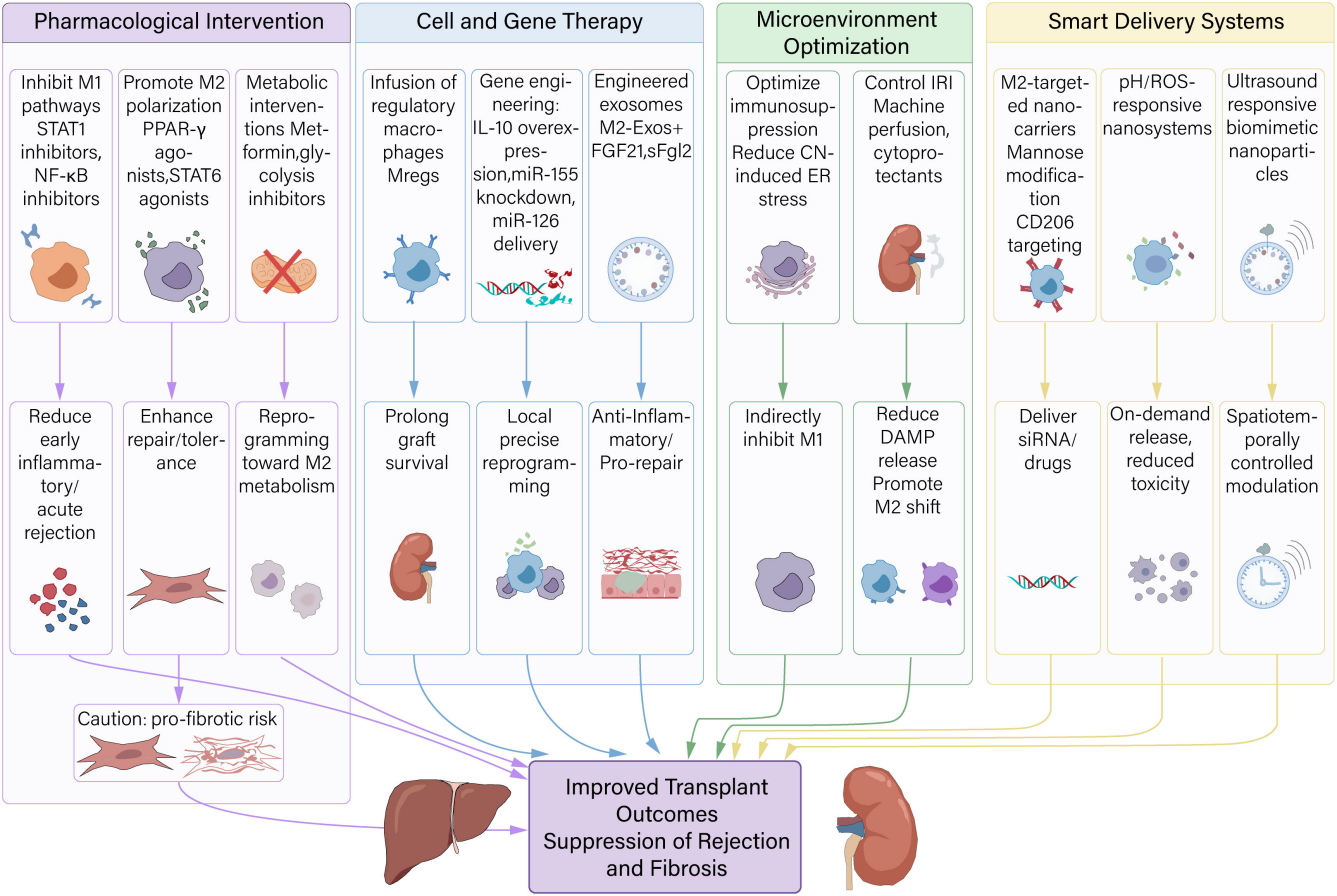
Multi-level therapeutic strategies targeting macrophage polarization to improve transplant outcomes. Pharmacological interventions include inhibiting M1-driving pathways (STAT1/NF-κB) to reduce acute rejection, promoting M2 polarization (PPAR-γ/STAT6 agonists) with caution regarding pro-fibrotic side effects, and metabolic reprogramming (e.g., metformin) to shift macrophages toward a repair phenotype. Cell and gene therapy approaches involve infusion of regulatory macrophages (Mregs), genetic engineering (IL-10, miR-155, miR-126), and engineered exosomes (M2-Exos carrying FGF21 or sFgl2) to locally modulate polarization. Microenvironment optimization aims to reduce IRI and ER stress, thereby indirectly favoring M2 over M1 polarization. Smart delivery systems, including mannose−modified M2−targeted nanocarriers, pH/ROS-responsive nanoparticles, and ultrasound-responsive biomimetic particles, enable spatiotemporally controlled and precise delivery of immunomodulatory agents. These combined strategies ultimately suppress rejection and fibrosis, leading to improved graft survival.

Controlling IRI is another crucial approach to improve the transplant microenvironment from the source and thereby indirectly regulate macrophage polarization. During IRI, the large amount of DAMPs released from cell damage, such as extracellular histone H3, acts as a key initial signal driving macrophage polarization towards a pro-inflammatory phenotype (121). Extracellular histone H3 can drive macrophages toward a pro-inflammatory phenotype by modulating HDAC2 expression and the subcellular localization of PKM2, thus accelerating the progression of acute liver failure (121). Hence, employing machine perfusion techniques or adding cytoprotective agents can effectively reduce IRI and decrease the release of DAMPs, thereby altering the initial signaling environment for macrophage polarization. For example, in liver transplantation, controlling IRI can reduce the polarization of Kupffer cells (liver-resident macrophages) toward the M1 phenotype and promote their conversion to the M2 phenotype, which has anti-inflammatory and tissue repair functions, thereby mitigating sterile inflammatory responses (122). Moreover, mathematical models predict that increasing the number of pro-regenerative macrophages within the system or accelerating their phenotypic switch can accelerate the clearance of senescent cells and tissue repair (123). These strategies collectively indicate that improving the transplant microenvironment by optimizing immunosuppressive regimens and controlling IRI can effectively guide macrophage transformation from a pro-inflammatory M1 phenotype to an anti-inflammatory, pro-repair M2 phenotype, thus playing a key role in reducing graft injury, promoting tissue repair, and inhibiting fibrosis, and offering new therapeutic targets for extending graft survival (**Figure 4**).

## 8 Future challenges and research directions

### 8.1 Beyond the M1/M2 dichotomy: macrophage heterogeneity at single-cell resolution

Traditionally, macrophage polarization has been simplified into the M1 (pro-inflammatory) versus M2 (anti-inflammatory/pro-repair) dichotomy model. However, this framework proves inadequate for explaining the complex and dynamic functions of macrophages following organ transplantation. With the application of high-throughput technologies such as single-cell RNA sequencing (scRNA-seq) and mass cytometry (CyTOF), researchers can now systematically map detailed macrophage atlases from human transplant specimens at single-cell resolution, revealing an astonishing degree of heterogeneity far beyond the M1/M2 paradigm. For example, in a study of placental chronic histiocytic intervillositis (CHI), multiplex immunofluorescence revealed that infiltrating macrophages predominantly exhibited a CD68+/CD206- phenotype, not simply classified by M1 or M2 categories (124). These cells interact with CD8+ T cells and carry an IFN-γ gene expression signature, with a molecular profile highly similar to renal transplant AMR, suggesting the existence of a specific macrophage subset associated with alloimmune injury (124, 125). Similarly, in a case of macrophage activation syndrome (MAS) occurring post-renal transplantation, glomerular infiltrating CD68+ foam cells were in frequent contact with CD8+ T cells, with the presence of activated CD8+ cells (CD8+, HLA-DR+). This unique cell interaction pattern also transcends the traditional M1/M2 framework (126).

Further research has revealed the trajectory of macrophage state transitions and their roles in specific pathological processes. After facial vascularized composite allotransplantation (VCA), a patient developed lymphadenopathy; histological examination showed numerous CD163+ histiocytes and dendritic cells within lymph nodes, accompanied by CD8+ T cell infiltration and granzyme B (GZMB) expression, suggesting an independent lymph node rejection episode (127). This finding indicates that within different microenvironments in the graft, macrophages may follow distinct differentiation paths to participate in specific pathological processes like vasculopathy or interstitial fibrosis. Furthermore, computerized image analysis of CHI showed that infiltrating macrophages strongly expressed the M2 marker CD163, which was not a simple anti-inflammatory phenotype but possibly an attempt to control inflammation, with its dysfunction paradoxically leading to persistent immune injury (128). Collectively, these studies indicate that novel subsets identified by single-cell techniques, such as CD68+/CD206- macrophages synergizing with CD8+ T cells, or CD163+ macrophages appearing in the fibrotic microenvironment, provide new directions for finding more specific therapeutic targets. For instance, intervention targeting the CXCR3 chemokine ligand system may reduce graft injury and fibrosis by blocking the recruitment of specific macrophage subsets (129). These findings clearly demonstrate that macrophage functional states in the transplant microenvironment form a continuous spectrum, and the simple M1/M2 label is insufficient to define pro−inflammatory or pro−repair/pro−fibrotic cells. In particular, newly identified pro−fibrotic subsets such as SPP1+ and TREM2+ macrophages challenge the conventional equation of ‘M2 = pro−fibrotic’ and explain why some therapeutic strategies targeting M2 polarization have yielded disappointing results. Future research should shift from ‘balancing M1/M2’ to ‘precisely targeting specific pathogenic subsets’, for instance, by blocking their recruitment (e.g., CCL2/CCR2 axis) or neutralizing their effector molecules (e.g., TGF−β, CTGF). Therefore, moving beyond the M1/M2 dichotomy to parse macrophage heterogeneity and functional states at the single-cell level is key to developing precise immunomodulatory strategies and improving long-term graft outcomes.

### 8.2 Spatiotemporal dynamics and fate determination studies

In the complex microenvironment of organ transplantation, the spatiotemporal dynamics and fate determination of macrophages are central to understanding the processes of graft injury, repair, and fibrosis. Macrophages of different origins, including yolk sac-derived tissue-resident macrophages (TRMs) and bone marrow-derived monocyte-derived macrophages (MDMs), exhibit distinct patterns of recruitment, proliferation, polarization, and turnover after transplantation. The development of intravital imaging technologies and genetic fate-mapping models provides unprecedented tools for studying these dynamic processes *in vivo*. For example, in CHI, a model considered maternal immune rejection of the semi-allogeneic fetus, studies found that infiltrating maternal macrophages mainly originate from peripheral blood monocytes rather than tissue-resident cells, with a predominantly CD68+/CD206- phenotype, indicating pro-inflammatory M1-like polarization characteristics (124). This mechanism is highly similar to that in solid organ transplant rejection, where bone marrow-derived macrophages massively infiltrate and mediate tissue injury (130). In contrast, under steady-state conditions, fetal Hofbauer cells in the placenta, as tissue-resident macrophages, primarily perform immunoregulatory and tissue repair functions, with a phenotype biased towards M2 (131). This difference in origin and function suggests that macrophages of different derivations may possess irreplaceable functional specificities in transplant injury, rejection, and repair.

Further exploration of the functional specificity of these different-origin macrophages is crucial for developing targeted therapeutic strategies. In transplant rejection, bone marrow-derived MDMs are typically recruited to inflammatory sites and, driven by the local microenvironment (e.g., IFN-γ), polarize into the M1 phenotype, releasing large amounts of pro-inflammatory factors and ROS, directly participating in tissue injury and acute rejection (115, 121). For example, in MAS occurring post-kidney transplantation, numerous CD68+ foam cells (histiocytes) within the glomeruli interact with CD8+ T cells, forming pathological changes similar to histiocytic glomerulopathy, directly demonstrating the critical role of bone marrow-derived macrophages in graft injury (126). Conversely, tissue-resident macrophages, such as Kupffer cells in the liver or alveolar macrophages in the lung, may maintain their population through self-renewal post-transplantation and tend to exert immunosuppressive and tissue repair functions. However, under sustained inflammatory stimulation, TRMs can also be reprogrammed, losing their protective phenotype and instead participating in chronic inflammation and fibrotic processes. For instance, in chronic rejection, TRMs may drive graft fibrosis by secreting pro-fibrotic factors like TGF-β and PDGF, promoting myofibroblast activation and ECM deposition (26). Therefore, clarifying the fate determination (e.g., proliferation, apoptosis, phenotype switching) of different-origin macrophages at various post-transplant stages and their contribution to graft outcome is a vital future research direction.

To parse these complex spatiotemporal dynamics *in vivo*, the integration of advanced imaging techniques and genetic tools is required. For instance, utilizing two-photon intravital imaging allows real-time observation of the migration of different-origin macrophages within the graft, their interactions with T cells, and their process of phagocytosing apoptotic cells. Combined with genetic fate-mapping models based on the Cre-loxP system, such as using Cx3cr1-CreER or S100a4-Cre reporter mice, yolk sac-derived TRMs or bone marrow-derived MDMs can be specifically labeled and tracked, enabling the dissection of their dynamic processes of proliferation, polarization, and turnover at the single-cell level (26). These studies will help answer crucial questions: In acute rejection, is the rapid infiltration of MDMs dominant in driving early injury, or is the activation and phenotypic switch of TRMs more critical? During chronic rejection and fibrosis stages, do TRMs maintain a pro-fibrotic cell pool through self-renewal? Furthermore, by comparing the dynamic changes of macrophages in different transplant models (e.g., kidney, heart, liver), we can reveal how organ-specific microenvironments influence macrophage fate decisions. Ultimately, breakthroughs in these basic research areas will provide a basis for developing spatiotemporally specific immune intervention strategies, such as blocking MDM recruitment or reprogramming the pro-fibrotic phenotype of TRMs, with the goal of promoting graft repair while suppressing rejection, thereby improving long-term graft survival.

### 8.3 Developing precise and intelligent targeted delivery systems

Given the dual role of macrophage polarization in organ transplantation, developing targeted delivery systems that can precisely regulate specific subsets is a frontier in current research. Designing nanocarriers that specifically recognize macrophage surface markers is key to achieving precise delivery. For example, M2 macrophages highly express the mannose receptor (CD206), while M1 macrophages highly express markers such as CD64. Studies have utilized mannose-modified microbubbles (MBman) to target CD206-positive M2 macrophages, enabling early non-invasive assessment of chronic rejection in heart transplantation through ultrasound molecular imaging, confirming the feasibility of CD206 as a target (132). Additionally, for M2-type TAMs, researchers have developed nanocarriers based on polyamidoamine (PAMAM) dendrimers grafted with D-glucuronic acid (Glu) to achieve CD206 targeting via mannose-mediated endocytosis, successfully delivering 2’3’-cGAMP to reprogram M2 macrophages into the anti-tumor M1 phenotype (133). In the field of organ transplantation, similar strategies could be used to precisely deliver immunosuppressive drugs or gene regulation tools (such as siRNA) to pro-fibrotic M2 macrophages within the graft, thereby inhibiting their pro-fibrotic function. For instance, mannose ligand-modified polymeric nanoparticles, targeting the macrophage mannose receptor (MMR), achieved efficient siRNA delivery to M2 macrophages for phenotypic reprogramming (134). These studies demonstrate that by recognizing specific surface markers, nanocarriers can achieve precise targeting of different macrophage subsets, providing a powerful tool for modulating the post-transplant immune microenvironment (135).

In addition to passive targeting, developing “smart” nanosystems that respond to environmental stimuli to achieve on-demand drug release, improving efficacy and reducing systemic toxicity, is another crucial direction. The post-transplant microenvironment possesses unique pathophysiological features, such as acidic pH in inflammatory areas, elevated levels of ROS, and enhanced activity of specific enzymes (like MMPs). Leveraging these features, nanocarriers can be designed to specifically release drugs at the lesion site. For example, a pH-responsive nanodelivery system based on β-cyclodextrin-poly(2-(diisopropylamino)ethyl methacrylate)/distearoylphosphatidylethanolamine-polyethylene glycol (β-CD-PDPA/DSPE-PEG) was able to release miR-223 in an acidic microenvironment, polarizing pro-inflammatory M1 macrophages into the anti-inflammatory M2 phenotype, effectively treating sepsis (136). In organ transplantation, IRI and acute rejection are often accompanied by local acidosis and oxidative stress, thus pH- or ROS-responsive nanocarriers hold great potential. For instance, a ROS-responsive nanocarrier containing thioketal bonds, capable of releasing 4-octyl itaconate and dexamethasone at the site of inflammation in response to M1 macrophage-produced ROS, reprogrammed M1 macrophages to the M2 phenotype for treating osteoarthritis (137). Applying such smart responsive systems to the transplant field holds promise for achieving localized, on-demand release of immunosuppressants within the graft, minimizing the risks of infection and malignancy associated with systemic immunosuppression. Furthermore, combining external physical stimuli, such as ultrasound, using sonoresponsive nanoparticles (e.g., M1/IR780@PLGA), can generate ROS under ultrasound irradiation, not only directly killing tumor cells but also inducing M2 macrophage-to-M1 transformation within the tumor microenvironment, activating anti-tumor immunity (138). Such multimodal intelligent delivery systems that combine endogenous microenvironment signals with exogenous physical stimuli open new pathways for developing safer and more effective post-transplant immunoregulatory strategies.

## 9 Conclusion

Macrophage polarization plays a decisive role in organ transplantation, with its regulatory network serving as the core hub connecting early injury to long-term prognosis. From an expert perspective, the field has evolved from a simple M1/M2 binary opposition model to a profound understanding of the high heterogeneity and plasticity of macrophages in the transplant microenvironment. M1 polarization acts as a potent engine of acute inflammation and rejection, and its overactivation is a key factor leading to primary graft nonfunction or early graft loss. Meanwhile, M2 polarization exhibits a complex duality, with its positive roles in tissue repair and immunoregulation coexisting alongside its negative roles in driving fibrosis and promoting chronic graft dysfunction. This requires that, when evaluating any therapeutic strategy targeting macrophages, we must move beyond a simplistic “good or bad” judgment and instead engage in refined spatiotemporal trade-offs.

Balancing the perspectives of different studies, the key lies in recognizing that macrophage function is not fixed but is dynamically shaped by the stage of graft injury, the local cytokine milieu, and interaction networks with other immune cells (such as T cells, dendritic cells). For example, inhibiting M1 polarization early in IRI may be beneficial, but during the acute rejection phase, completely suppressing all inflammatory responses might compromise necessary anti-pathogen defenses. Similarly, promoting M2 polarization during the repair phase aids tissue healing, but if unchecked and sustained, it sows the seeds for interstitial fibrosis. Therefore, the future therapeutic direction should not be global “inhibition” or “promotion” of a particular polarization state, but rather the development of “context-dependent” precision interventions.

Currently, pharmacological interventions, cell therapies (such as infusion of regulatory macrophages), and genetic engineering approaches, although promising, face significant challenges in clinical application. The core challenges lie in achieving target specificity, avoiding systemic interference with host defense and homeostasis maintenance, and precisely grasping the therapeutic time window. Cutting-edge technologies such as single-cell sequencing, spatial transcriptomics, and intravital imaging are providing us with unprecedented tools to map the differentiation trajectories and spatial distribution dynamics of macrophages within grafts. Based on these maps, the development of nanocarriers or engineered cells capable of responding to local microenvironmental signals and intelligently modulating macrophage functional states will be key to achieving the leap from “crude regulation” to “precision reprogramming”.

In summary, through an in-depth understanding of the complex logic of macrophage polarization and the development of spatiotemporally precise regulatory strategies, we are poised to transform this key immune node into a powerful therapeutic lever. This can break the vicious cycle of post-transplant injury-fibrosis, ultimately advancing organ transplantation from the goal of merely improving short-term survival to the higher objective of ensuring long-term healthy survival and robust function of grafts.
